# Antipsychotic drug use and risk of stroke and myocardial infarction: a systematic review and meta-analysis

**DOI:** 10.1186/s12888-019-2177-5

**Published:** 2019-06-20

**Authors:** Sanja Zivkovic, Chan Hee Koh, Nandita Kaza, Caroline A. Jackson

**Affiliations:** 1 Usher Institute of Population Health Sciences & Informatics, University of Edinburgh, Nine Bioquarter, 9 Little France Road, Edinburgh, EH16 4UX Scotland; 2 College of Medicine and Veterinary Medicine, University of Edinburgh, Chancellor’s Building, 49 Little France Crescent, Edinburgh, EH16 4SB Scotland

**Keywords:** Antipsychotics, Myocardial infarction, Stroke, Dementia, Meta-analyses

## Abstract

**Background:**

The effect of antipsychotic (AP) drugs on risk of stroke and myocardial infarction (MI) remains unclear due to methodological limitations of, and inconsistencies across, existing studies. We aimed to systematically review studies reporting on the associations between AP drug use and stroke or MI risk, and to investigate whether associations differed among different sub-populations.

**Methods:**

We searched Medline, EMBASE, PsychINFO and Cochrane Library (from inception to May 28, 2017) for observational studies reporting on AP drug use and MI or stroke occurrence. We performed random-effects meta-analyses for each outcome, performing sub-groups analyses by study population – specifically general population (i.e. those not restricted to patients with a particular indication for AP drug use), people with dementia only and psychiatric illness only. Where feasible we performed subgroup analyses by AP drug class.

**Results:**

From 7008 articles, we included 29 relevant observational studies, 19 on stroke and 10 on MI. Results of cohort studies that included a general population indicated a more than two-fold increased risk of stroke, albeit with substantial heterogeneity (pooled HR 2.31, 95% CI 1.13, 4.74, I^2^ = 83.2%). However, the risk among patients with dementia was much lower, with no heterogeneity (pooled HR 1.16, 95% CI 1.00, 1.33, I^2^ = 0%) and there was no clear association among studies of psychiatric populations (pooled HR 1.44, 95% CI 0.90, 2.30; substantial heterogeneity [I^2^ = 78.8])). Associations generally persisted when stratifying by AP class, but few studies reported on first generation AP drugs. We found no association between AP drug use and MI risk (pooled HR for cohort studies: 1.29, 95% CI 0.88, 1.90 and case-control studies: 1.07, 95% CI 0.94, 1.23), but substantial methodological and statistical heterogeneity among a relatively small number of studies limits firm conclusions.

**Conclusions:**

AP drug use may be associated with an increased risk of stroke, but there is no clear evidence that this risk is further elevated in patients with dementia. Further studies are need to clarify the effect of AP drug use on MI and stroke risk in different sub-populations and should control for confounding by indication and stratify by AP drug class.

**Electronic supplementary material:**

The online version of this article (10.1186/s12888-019-2177-5) contains supplementary material, which is available to authorized users.

## Background

Major mental disorders represent a growing and, until relatively recently, under-recognized global public health burden. Schizophrenia, bipolar disorder, major depression and anxiety feature among the top 20 causes of years lived with disability (YLD) [1] and are associated with marked premature mortality [2–4]. Much of this excess mortality is due to a higher burden of cardiovascular and cerebrovascular disease (largely ischaemic heart disease and stroke) compared to the general population [5–7]. Several factors related to mental illness, including low socioeconomic status, lifestyle, physical comorbidities, genetic predisposition and healthcare access [7–9] could contribute to increased cardio- and cerebrovascular disease incidence in this vulnerable group. Prescription medication, including antipsychotic (AP) drug use, has also been raised as a possible cardiovascular and cerebrovascular disease risk factor, potentially operating through effects on body weight, metabolic factors and thrombosis [10].

Despite their potential to increase risk of circulatory disease, AP drugs are also being increasingly used off-label, for the treatment of dementia, anxiety, insomnia and post-traumatic stress [11], with little understanding of the long-term side-effects, including risks of major cardiovascular events such as myocardial infarction and stroke. Their increased use in patients with dementia is particularly concerning, with around 20% of patients with dementia in nursing homes in the USA and UK treated with APs [12, 13]. Use of these drugs in this population has been linked to increased risk of stroke as well as other adverse outcomes, including increased mortality [14] and thus regulatory bodies have discouraged the use of AP drug use in people with dementia [15]. However, the evidence for increased stroke risk is based largely on analyses of serious adverse events in randomized controlled trials of AP drugs in patients with dementia [10, 16, 17]. Confirmation of the appropriateness of these attached warnings to AP drug use is important, given that pharmacological alternatives for the treatment of behavioral and psychological symptoms of dementia are quite limited [18].

A number of observational studies have reported on the association between AP use and stroke risk, but until very recently these had not been systematically reviewed. Since commencing the present review, one systematic review and meta-analysis has been published which concluded that first generation, but not second-generation AP drug use was associated with an increased risk of cerebrovascular disease. The authors also reported that among those with dementia in particular, use of any AP was associated with a low risk of cerebrovascular disease [19]. However, this study identified almost half as many studies as in the present review, thus omitting relevant additional studies on this topic. Earlier reviews focused on summarizing the effects of AP drug use specifically in the elderly or those with dementia [10, 20], the most recent of which suggests that AP drug use may be associated with an increased risk of stroke in this population [20]. Existing systematic reviews on the association between AP drug use and risk of myocardial infarction (MI) are conflicting. The first review concluded that the evidence for an association is inconclusive [21], whereas a more recent review concluded that AP drug use is associated with an increased risk of MI [22]. Interestingly, a subsequent additional review (which was published after we completed the screening phase of the present review) included the same studies as this previous review, but drew different, more cautious, conclusions [23]. There is therefore a lack of clarity on the associations between AP drug use and the occurrence of major cardio- and cerebrovascular events. To address this, we performed a systematic review and meta-analysis of studies reporting on the association between antipsychotic drug use and risk of stroke and MI.

## Methods

### Search strategy

We searched Embase, MEDLINE, PsychINFO (via OVID) and the Cochrane Library from their origin to May 28, 2017 using a comprehensive search strategy comprising medical subject heading terms and free text words for the exposure (AP drugs) and outcomes (stroke and MI) of interest (Additional file 1). We restricted our search to English language articles only and perused reference lists from previous reviews and relevant included studies to identify any additional studies. The first reviewer (SZ) and one of 2 second reviewers (NK and AK) independently screened all titles and abstracts, and, for potentially relevant studies, screened full text articles to determine eligibility for inclusion. We included conferences abstracts that were indexed in the search engines.

### Inclusion and exclusion criteria

We sought studies that included a general population (i.e. those not restricted to patients with a particular indication for AP drug use) or people with specific mental disorders often treated with AP drugs (e.g. schizophrenia, bipolar disorder, major depression or dementia), and compared AP drug use versus no AP drug use in relation to risk of stroke or MI. We anticipated that confounding by indication [24] could be a limitation of many studies and so we did include studies which attempted to address this by comparing people taking APs versus those on other medications for mental illness (but not taking APs). Confounding by indication could occur because the indication for AP drug use, such as having a serious mental illness, is itself associated with increased risk of stroke or MI, thus potentially leading to a spurious association between AP drug use and stroke or MI risk. We excluded studies: that compared AP drug use with active comparators; and that reported only on stroke or MI mortality, since we were interested in the association between AP drug use and risk of stroke/MI occurrence and not death following stroke/MI. Where two studies overlapped in terms of study population we selected the larger of the two studies.

### Data extraction

Pairs of reviewers (SZ and CK or SZ and NK) independently extracted data on: study design; sample size; country; patient population; baseline characteristics of each comparator arm; definition of exposure and outcome; ascertainment of exposure and outcome; length of and loss to follow up; number of events; main results including crude and adjusted effect estimates with accompanying 95% confidence intervals; method of statistical analysis; and adjustments for confounders. A fourth investigator (CAJ) reviewed any disagreements. We assessed study quality and risk of bias using the SIGN checklist [25].

### Meta-analysis

We used Stata version 14.0 to conduct meta-analyses on the association between AP drug use and each of stroke and MI occurrence. We pooled studies according to study design and type of effect estimate, grouping together: cohort and nested case-control studies reporting hazard ratios (HRs); cohort/nested case-control studies reporting odds ratios (ORs); and standard case-control studies reporting ORs. For stroke, we stratified these meta-analyses by type of study population, creating sub-groups of studies that included: patients diagnosed with psychiatric disorders; patients diagnosed with dementia; and a general population with unspecified indications for AP use. Whilst we summarized findings from self-controlled case series and case-crossover studies, we did not include these studies in our meta-analyses, given the differences in study design and analytical approach. Due to a fewer number of studies, almost all of which were conducted using general population data, we were unable to perform sub-group analysis by type of study population when pooling together studies reporting on MI. We pooled effect estimates using the random effects method, assessing heterogeneity using the I^2^ and Chi^2^ statistics. In accordance with Cochrane Collaboration guidance, we interpreted heterogeneity based on I^2^ values as follows: 0–40% - may not be important; 30–60% - may be moderate; 50–90% - may be substantial; 75–100% may be considerable. We interpreted I^2^ values in the context of the magnitude and direction of effects as well as the chi^2^ *p*-value for heterogeneity. In order to improve comparability between studies, where results on the association between AP drug use and cerebrovascular disease risk were reported for different exposure periods we included the longest exposure period, often defined as the ‘ever exposed’ period, or as close to is as possible. We narratively summarized findings from self-controlled studies, since it is methodologically inappropriate to pool this type of study design with cohort or case-control studies. In addition, methodological differences (including the reporting of different types of effect estimates) precluded separate pooling of these studies. Where outcome risks by type of AP drug were reported (i.e. first generation antipsychotic [FGA] or second generation antipsychotic [SGA]), we included these separately in the analyses, thus obtaining sub-group summary effect estimates for type of AP as well as overall summary estimates. If studies reported on multiple APs separately, we included the largest group of AP users in our meta-analysis.

We reported the findings of this systematic review in accordance with the PRISMA and MOOSE guidelines [26, 27].

## Results

**Fig. 1:**
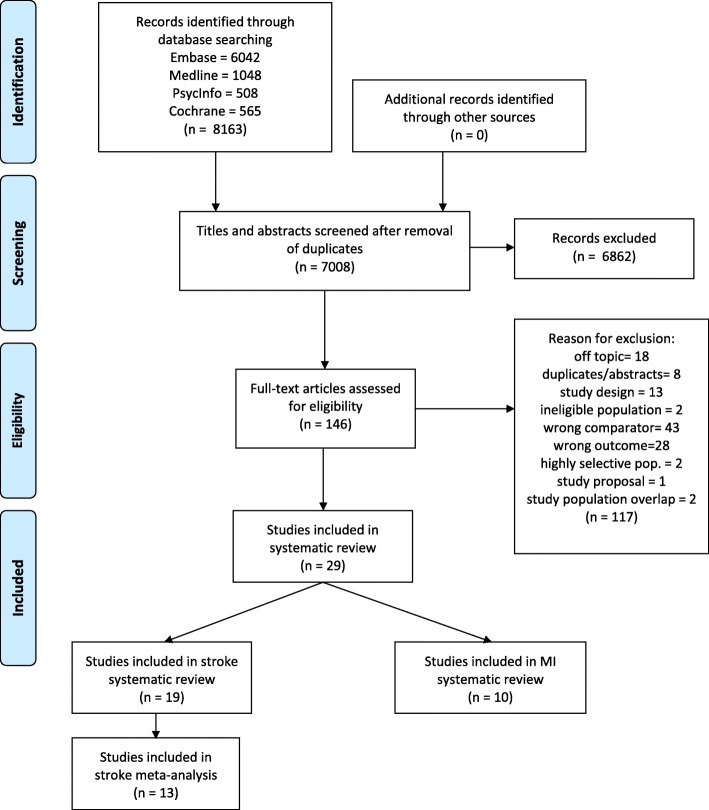
Flow diagram of literature search and study selection

Our search strategy identified 8163 articles. After de-duplication we screened 7008 titles and abstracts, 146 of which were potentially relevant. Following full-text review of these articles, we included 19 articles reporting on cerebrovascular disease [28–46] and 10 reporting on MI [31, 47–55] in the review (Fig. 1).

### Quality assessment of included studies

All but three studies [38, 53, 54] used routine administrative health datasets to ascertain AP prescribing and stroke/MI ascertainment, thus eliminating reporting bias and minimizing loss to follow-up (Additional file 1: TablesS1 and S2). The remaining three studies relied on self-reported AP drug use and/or outcome occurrence. Twelve studies reporting on stroke and 7 reporting on MI reported baseline characteristics of comparison groups. All studies adjusted for age and sex and the majority also accounted for cardiovascular comorbidities. Among studies that included a general population (e.g. a cohort identified from primary care/medical insurance records), indication for AP drug use was generally not accounted for. One study compared stroke and MI outcomes in people taking SGAs versus a comparison group taking anti-depressants but not SGAs, in order to partially control for confounding factors associated with initiation of SGAs (related to mental illness) [31]. Socioeconomic status and lifestyle factors, which may be associated with the disorder for which AP drug medication was prescribed, were rarely adjusted for (Additional file 1: Tables S3 and S4). We identified one conference abstract by Wang et al. which was assessed to be of a reasonable quality overall. However, we couldn’t assess some elements due to the lack of available information [55].

### Antipsychotic drug use and stroke risk

Eight cohort studies [28, 29, 31, 36, 38, 40, 41, 43], 6 case-control studies (5 nested case-control) [30, 33–35, 37, 39], two self-controlled case series studies [32, 42], two case-crossover studies [44, 46] and one case-case-time control study [45] reported on the association between AP drug use and stroke risk. Five studies included a population of dementia patients [28, 29, 37, 39, 40], three included patients with psychiatric disorders [30, 34, 36] and the remaining 11 studies investigated AP drug use in a general population (Table 1). All but one [38] of the included studies used large routine medical databases to identify patients, determine AP drug use and identify outcome. AP drug exposure was defined as new AP drug use [28, 29, 42, 43] or ‘ever’ AP drug use. Only 3 studies specified a minimum exposure period, which ranged from 3 days to 4 weeks [31, 33, 45].

**Table 1:** Characteristics of included studies, stratified by outcome

Author, year, country	Study design	Population	Sample size^a^	Mean age (years)	Male (%)	Length of follow up	Antipsychotic drug use definition	Case or outcome Definition
**STROKE**								
Barnett, 2007, [28] USA	Cohort	Veterans with dementia aged ≥65 years, identified between 2002 and 2003 from the Veterans Administration and Medical Provider and Analysis Review database	14,029	78	97	1.5 years	New AP drug use (excluding clozapine and injectable APs)	Hospitalisation for or death from CVE
Chan, 2010, [29] China	Cohort	Hong Kong residents with dementia aged ≥65 years, identified between 2000 and 2007 from the Pamela Youde Nethersole Eastern Hospital	1089	81	34	1019 days	New AP drug use	Hospitalization for CVE
Chen, 2008, [30] USA	Nested Case-control	People with depression and without a history of stroke between 1998 to 2002, identified from the PHARMetrics database	7601	NR(66.4% aged 35–64)	37	Minimum of 6 months	Any use of risperidone (SGA)	First diagnosis of stroke
Correll, 2015, [31] USA	Cohort	People aged 18–65 years without a history of hypertension, diabetes, obesity or CHD, identified between 2006 and 2010 from Thomson Reuters MarketScan Research insurance database	284,234	44.5	29	1.5 years	Any SGA use for at least 4 weeks	First diagnosis of stroke or TIA (from inpatient and outpatient records)
Douglas, 2008, [32] UK	SCCS	People registered with a GP in England/Wales, AP prescribed 1988–2002; identified from the General Practice Research Database	6790	80	36	0.4 years	Any AP use	First stroke diagnosis (no ICD codes reported)
Franchi, 2013, [33] Italy	Case control	Community dwelling seniors of Lombardy aged 65–94 years between 2003 and 2005; identified from the Lombardy Regional Databases	19,275	NR	46	NR	≥2 boxes of any AP dispensed	Hospitalisation for stroke
Hsieh, 2013, [34] Taiwan	Nested Case-control	People with schizophrenia identified between 2001 and 2009 from the National Health Insurance Research Database	1158	57.3^b^	50	957^b^ days	Any AP use	First diagnosis of stroke^c^ or TIA (from inpatient and outpatient records)
Kleijer, 2009, [35] Netherlands	Nested Case-control	Community dwelling adults aged > 50 years, starting an antipsychotic between 1986 and 2003 and identified from the national prescription, vital statistics and health registries databases	2548	76	44	Minimum of 8 years	Any AP use	Hospitalisation for Stroke^c^ or TIA
Lan, 2015, [36] Taiwan	Cohort	People with bipolar disorder identified between 2001 and 2006 from the National Health Insurance Research Database	3681	43	42	11 years maximum	Any AP use	First hospitalisation for (or death from) stroke^c^ (not including TIA)
Laredo, 2011, [37] UK	Nested Case-control	People aged ≥65 years diagnosed with dementia and GP registered in 1995, identified from the General Practice Research Database	18,762	81^b^	30^b^	12 years	Any AP drug use	First stroke diagnosis^c^
Liebetrau, 2008, [38] Sweden	Cohort	85 year olds in community/institutions registered with Gothenburg census who completed survey and neuro-psychiatric exam 1986–87	401[281]	85	30	3 years (29% loss to follow up)	Any AP drug use	First diagnosis of stroke^c^
Liperoti, 2005, [39] USA	Nested Case-control	Nursing home residents aged ≥65 years between 1998 and 1999, diagnosed with dementia and identified from the Systematic Assessment of Geriatric drug use via Epidemiology database	4788	NR(50.7% aged > 85)	29^b^	Maximum of 3 months	Any AP use within the 3 months prior to the index date	Hospitalisation for ischemic stroke or TIA
Liu, 2013, [40] Taiwan	Cohort	People aged ≥65 years, diagnosed with dementia between 2003 and 2005, identified from the Longitudinal Health Insurance Database 2005	8957 [2243]	78	47	2.6 years	Any AP use	First diagnosis of CVE
Percudani, 2005, [41] Italy	Cohort	Residents of Lombardy region between 2001 and 2002 aged ≥65 years, identified from Lombardy regional databases	1,645,978	NR	NR	1 year	Any AP use during 2001	Hospitalisation for stroke^c^ or TIA
Pratt, 2010, [42] Australia	SCCS	Veterans aged ≥65 years, with stroke diagnosis between 2003 and 06, identified from the Australian Department of Veterans Administration claims database	10,638	84	NR	3–4 years	New AP drug use (excluded if using FGA and SGA together or injectable APs)	Hospitalization for stroke^c^
Sacchetti, 2008, [43] Italy	Cohort	People aged ≥65 years between 2000 and 2003, identified from the Health Search Database	74,162	76^b^	42^b^	maximum of 4 years	New AP drug use	First diagnosis of stroke
Shin, 2013, [44] Korea	Case Crossover	People aged 65–100 years diagnosed with a stroke between 2005 and 2006; identified from the Korean Health Insurance Review and Assessment Service	1601	76	42	150 days	Any SGA use (respiridone, quetiapine or olanzapine) within the 30-day case and 60-day control period	First hospitalisation for ischemic stroke
Wang, 2012, [45] USA	Case-case-time control	Veterans diagnosed with a stroke between 2002 and 2007, with one physician visit and prescription in the year before hospitalization; identified from the Veterans Health Administration database	511	NR (69.1% were aged 60–90)	98^b^	120 days	Any AP use with at least 3 days duration in 30-day case or control periods	First hospitalization for ischaemic stroke
Wu, 2012, [46] Taiwan	Case Crossover	People aged ≥18 years with incident stroke between 1998 and 2007, identified from the National Health Insurance Research Database	14,584	69	49	1 year	Any AP use in year prior to stroke (excluding prochlorperazine, melitracen-flupentixol and injectable APs)	First hospitalisation for stroke^c^ or TIA
MYOCARDIAL INFARCTION								
Brauer*,* 2015, [47] UK	Case-control	People aged ≥18 years and enrolled with a GP between 1987 and 2010, identified through the Clinical Practice Research Datalink database	136,095	67^b^	68^b^	Minimum of 1 year	Any current AP use within 90 days of outcome	MI diagnosis
Correll, 2015, [31] USA	Cohort	People aged 18–65 years enrolled between 2006 and 2010 by Thomson Reuters MarketScan Research insurance database	284,234	45	29	1.5 years	Use of any SGA for at least 4 weeks	MI diagnosis
Enger, 2004, [48] USA	Cohort	People aged 15–64 years enrolled in United Healthcare between 1995 and 1999, diagnosed with schizophrenia and being treated with antipsychotics, matched to general population controls identified from an affiliated research database	11,520	40	42	1.5 years	Any AP use	Hospitalization for MI
Hwang*,* 2014, [49] Canada	Cohort	Community and long-term care residents in Ontario, aged > 65 years between 2003 and 2011, identified from linked Ontario public prescription, vital statistics and health registries	195,554	81	36	90 days	Any new SGA use (quetiapine, olanzapine and risperidone)	Hospitalization for MI
Lin, 2014, [50] Taiwan	Case Crossover	People aged ≥18 years with schizophrenia, mood or dementia disorder, prescribed antipsychotics and who had a first AMI between 1999 and 2009, identified from the Taiwan’s National Insurance Research Database	56,910	72	52	120 days	Any AP use	First MI hospitalization
Nakawaga, 2006, [51] Denmark	Case-control	Danish residents aged ≥15 years, identified 1992 to 2003 from the Danish Civil Registry and Medical Database	128,262	70	61	1 year	Any current AP use (within 90 days prior to index date)	Hospitalization for MI
Pariente*,* 2012, [52] Canada	Cohort	Community-dwelling residents with dementia and aged > 65 years, in Quebec, Canada, identified 2000–2009 from public prescription and medical service database	21,938	NR(53% aged > 79)	34	Maximum of 1 year	Any new AP use	Hospitalisation for MI
Penttinen, 1996, [53] Finland	Case-control	Finnish farmers born between 1935 and 1958; without a history of CVD and recruited between 1980 and 1992, with data collected from questionnaires and medical records	332	NR (Range: 22–57)	100	100	Any AP use	First MI hospitalization/ death from MI
Pratt, 1996, [54] USA	Cohort	Baltimore residents aged > 18 years without a history of ‘heart trouble’, recruited via household interviews	1551	NR(67% aged < 45)	37	13 years (28% loss to follow up)	Any use of SGA	First non-fatal MI (self-report)
Wang, 2011, [55] USA	Case-case time control	Veterans aged 50–90 years, identified between 2002 and 2007 from the Veterans Health Database	NR	NR	NR	120 days	NR	Hospitalization for MI

^a^Size of subsample actually included in analyses, where relevant

^b^Weighted average calculated by review authors based on information provided in the article

^c^Based on broad stroke definition which includes all cranial haemorrhages and non-acute and non-acute cerebrovascular disease

*AMI* acute myocardial infarction, *AP* antipsychotic, *CHD* coronary heart disease, *CVE* cerebrovascular event, *FGA* first generation antipsychotic, *GP* general practitioner, *MI* myocardial infarction, *NR* not reported, *SGA* second generation antipsychotic, *TIA* transient ischaemic attack

The mean baseline age of study participants varied by the study populations included (Table 1). The mean age in studies that included people with dementia only ranged from 78 to 81 years, whilst studies of patients with psychiatric conditions had a younger range (43 to 57 years). The age range in studies that included a general population was much broader (45 to 85 years), reflecting different age restrictions and data sources used. Studies were heterogeneous in terms of follow-up duration, ranging from 1 week [42] to 12 years [37]. Among studies that included a general population, the indication for AP drug use was not reported.

**Fig. 2:**
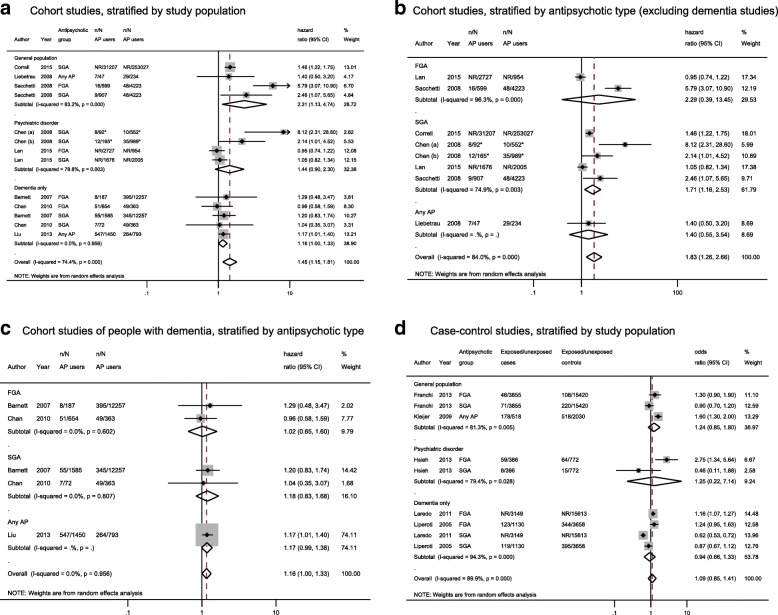
Forest plots for the association between antipsychotics and stroke, by study population/antipsychotic class subgroups NB In plots a-c, n/N represents number of cerebrovascular events/total number in comparison group for those using antipsychotic medication and those not using antipsychotic medication. Chen (a) = haemorrhagic stroke; Chen (b) = ischaemic stroke. AP = antipsychotic; CI = confidence interval

We included all but one [41] of the cohort and case-control studies in our meta-analysis. We separately pooled 13 study populations from 8 cohort studies (including one nested case-control study reporting HRs) [28–31, 36, 38, 40, 43] and 9 study populations from 5 case-control studies [33–35, 37, 39]. Overall, among studies including a general population, use of any AP drug was associated with a 2.3-fold increased risk of stroke (pooled HR 2.31, 95% CI 1.14, to 4.74), but with substantial heterogeneity between studies (I^2 =^ 83.2%; *p* < 0.001; Fig. 2a). This appeared to be due to larger effect estimates in the study which examined new (as opposed to ‘ever’) AP drug use [43]. Findings were more mixed for studies including only psychiatric patients, where use of any AP drug was associated with a statistically non-significant increased risk of stroke (pooled HR 1.44, 95% CI 0.90 to 2.30), with substantial heterogeneity between studies (Fig. 2a). Similar findings were observed when we pooled case-control studies, although effect estimates were weaker and not significant (Fig. 2d). When we pooled studies that included a general population or patients with psychiatric conditions and stratified by AP drug use, SGA drug use was significantly associated with a 71% increased risk of stroke (pooled HR 1.71, 95% CI 1.16 to 2.53; Fig. 2b), again with substantial heterogeneity between studies (I^2^ = 74.9%, *p* = 0.003). Only two studies reported on FGA drug use, with contradictory findings (Fig. 2b).

Findings between studies of patients with dementia were more consistent. Use of any AP drug was marginally significantly associated with a 16% increase risk of stroke among cohort studies (pooled HR 1.16, 95% CI 1.00 to 1.33), with no heterogeneity between studies (Fig. 2a). Findings were consistent across studies, irrespective of whether AP drug use was defined as ‘new’ or ‘ever’. When dementia studies were stratified according to AP type, neither FGA nor SGA drug use were statistically significantly associated with stroke risk (pooled HR 1.02, 95% CI 0.65 to 1.60 and pooled HR 1.18, 95% CI 0.83 to 1.68, respectively; Fig. 2c), however this is based on just two studies in each sub-group. In keeping with findings from cohort studies, findings from case-control studies showed no clear association between AP drug use and stroke risk in studies of patients with dementia (pooled OR 0.94, 95% CI 0.66 to 1.33), but there was substantial heterogeneity between studies (I^2^ = 94.3%, *p* < 001; Fig. 2d).

An additional five self-controlled studies (among which three different variations of the self-controlled approach were adopted) [32, 42, 44–46] compared pre- and post-periods of AP drug use for each patient. All five studies found AP drug use was associated with an increased risk of stroke, although follow-up was much shorter than in other study designs, ranging from 1 week to 1 year. Three found a significant association between use of any AP drug and stroke [32, 45, 46]. Shin found a significant association between SGA drug use and increased stroke risk (OR 3.90, 95% CI 3.30 to 4.60) [44] while Pratt reported a significant association between FGA drug use (but not SGA drug use) and increased stroke risk during the week following AP drug initiation (IRR 2.25, 95% CI 1.32 to 3.83) [42]. These findings are also consistent with those from Sacchetti, which found a substantial increased risk of stroke within one-month post-AP drug use initiation [56]. This study wasn’t included in our review since the study population overlapped with that of another study already included [43].

### AP drug use and MI risk

Five cohort studies [31, 48, 49, 52, 54], three case control studies [47, 51, 53], one case crossover study [50] and one case-case time control study [55] reported on AP drug use and MI risk (Table 1). Seven studies included a general population and thus did not specify the indication for AP drug use [31, 47, 49, 51, 53–55], one included patients with schizophrenia [48], one included patients with psychiatric illness or dementia disorders [50] and one included patients with dementia [52]. In five studies the included population had a mean age of more than 65 years, four included a population aged < 50 years, and one study did not report on age of participants. The follow-up period ranged from 90 days [49] to 13 years [54].

**Fig. 3:**
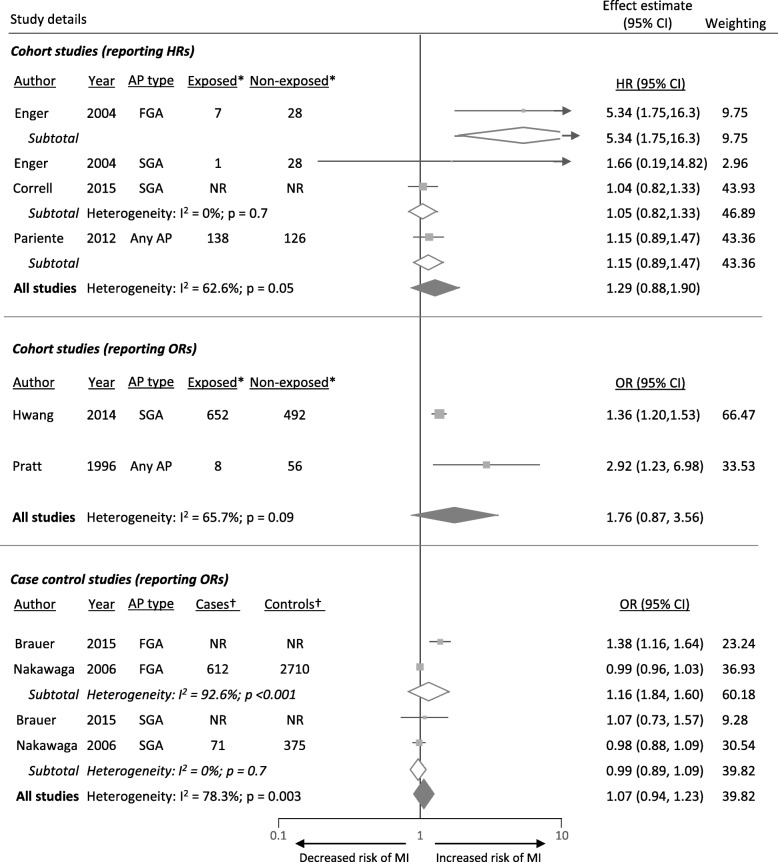
Forest plots for the association between APs and MI, by study design and AP class. *Number of MI events in the group prescribed antipsychotic drugs (exposed) and those not prescribed antipsychotic drugs (non-exposed). †Number of people prescribed antipsychotic drugs among cases (those with MI) and controls (those without MI). AP = antipsychotic; HR = hazard ratio; MI = myocardial infarction; NR = not reported

Study findings among five cohort studies were inconsistent, with substantial heterogeneity between studies reporting HRs (I^2^ = 62.6%; *p* = 0.05) and those reporting ORs (I^2^ = 65.7; *p* = 0.09). There were too few studies to allow sub-group analysis by type of study population and a limited sub-group analysis by AP drug type (Fig. 3).

Similarly, there was considerable heterogeneity between the findings from case-control studies (I^2^ = 78.3%; *p* = 0.003). Given this statistical heterogeneity, along with methodological heterogeneity in terms of study population and type of AP drug included, summary estimates should be interpreted with caution. Based on existing studies, there was no clear evidence that AP drug use was associated with increased risk of MI (Fig. 3).

In contrast to the findings from cohort and case-control studies, findings from the two self-controlled studies were consistent in showing a significant association between AP drug use and increased risk of MI (50,55). As with the self-controlled studies that examined stroke outcomes, these studies differ from cohort and case-control studies in terms of including a much shorter follow-up period (30–120 days; Table 1).

## Discussion

We found that AP drug use is associated with an increased risk of stroke, but that this association is much weaker in studies that included only patients with dementia than in studies which included the general population or patients with psychiatric disorders. Among study populations that were not restricted to patients with dementia, an increased stroke risk was observed for SGAs only, with less available data on FGAs. However, with the exception of the subgroup of dementia-focused studies, in all analyses there was substantial statistical heterogeneity between studies, even after stratifying by study population and type of AP drug.

There was no clear evidence of an association between AP drug use and risk of MI. However, substantial statistical and methodological heterogeneity between among a relatively small number of studies on MI risk makes it difficult to draw firm conclusions.

We are aware of just one other systematic review and meta-analysis on AP drug use and stroke risk, published during the conduct of our review [19]. This review included fewer studies than we did, but this is partly due to our use of a broader set of inclusion criteria. Whilst the authors of the previous review did perform sub-group analyses, these were not identical to those that we performed, which makes comparability of findings difficult. Consistent with the findings of our review, the authors did report a weaker association between AP drug use and stroke risk among the dementia population compared to studies not restricted to this sub-population. We found that AP drug use was associated with a 16% increased odds of stroke in those with dementia, which was similar to the 17% (OR 1.17, 95% CI 1.08 to 1.26) reported by Hsu and colleagues [19]. Previous narrative reviews drew similar conclusions, but focused on summarizing evidence on AP use and stroke risk in the elderly or those with dementia [10, 20]. Interestingly, Sacchetti concluded that the excess cerebrovascular disease risk does not appear to be confined to patients with dementia, but applies to elderly patients in general [20]. In contrast to our findings, Hsu et al. concluded that the association with stroke risk is stronger for FGAs than SGAs [19]. Conversely, although relatively few studies reported findings by AP drug type, we found more convincing evidence of an association between SGAs and stroke risk, with more limited data available for FGAs. However, there are a number of key differences between the two reviews, which may account for these differing conclusions. In addition to including a greater number of studies, we also stratified by study design in all analyses. In contrast, Hsu et al. appeared to have pooled studies irrespective of study population, type of study and type of effect estimate. Interestingly, a recent meta-analyses found no difference in risk of cerebrovascular events in those prescribed FGAs versus SGAs when pooling data from 5 studies that directly compared the effect of FGAs versus SGAs on cerebrovascular disease risk in people with dementia [57].

As with stroke, studies on MI were heterogeneous in terms of study design, AP drug type, study population and measure of effect. Although we found no clear evidence that AP drug use is associated with increased MI risk, the inconsistency in findings across studies, in the presence of substantial statistical heterogeneity, makes it difficult to draw firm conclusions. This concurs with the findings of a previous review by Brauer [21], despite our review having included an additional 5 studies. Our conclusion contradicts that of a more recent review, which concluded that there is an association between AP drug use and MI risk, albeit with substantial heterogeneity between pooled effect estimates [22]. We identified two additional studies not included in this review [31, 49]. Whilst the authors of this review included sub-group analyses, they still pooled results from methodologically different studies or different study sub-populations and often pooled different effect estimates, which affects reliability and interpretability of pooled results. This may account for the contradiction in findings between the present review and this previous review. Our conclusions do however concur with those of Huang et al., who performed a similar review concurrent to our own review [23]. In line with our findings, the authors conclude that there is insufficient evidence to draw firm conclusions about the association between APs and MI risk and highlight the need for further prospective studies in this area. Given that the conclusions drawn by Yu et al. and Huang et al. are based on the same included studies, but are inconsistent, our review makes an important further addition to the literature, providing support for the conclusions and recommendations by Huang et al. In our review we did not synthesize data on differing time periods of AP drug exposure and risk of stroke and MI. Effect estimates for different AP drug exposure periods were reported in relatively few studies, which were extremely methodologically heterogeneous, limiting formal synthesis. However, within individual studies, there is some evidence that the risk of stroke and MI might be greater in the very acute period of AP drug use, with a lower risk in the longer term. This is consistent with findings from other reviews [20, 22, 23]. These findings largely stem from studies that included a general, older population and so it is unclear whether such a pattern is also present amongst people with major psychiatric disorders for which AP drugs are indicated. Given that relatively few studies have investigated this across different sub-populations, further careful investigation in future studies is needed.

There is biological plausibility for a causal association between AP drug use and increased risk of vascular disease. AP drug use has long been associated with weight gain, which is possibly due to the AP drug affinity for the histamine-1 receptor which, when blocked, modulates feeding behavior and stimulates weight gain [58, 59] and the effect of AP drugs on hypothalamic regulation [60, 61]. AP drug use is also associated with risk of metabolic syndrome, perhaps through the increased affinity for specific serotonin, muscarinic and histamine receptors, among others [62]. APs may also increase risk of thrombosis. A recent meta-analysis [63] found that AP drug use is associated with a 50% increased odds of venous thromboembolisms, albeit with substantial heterogeneity between studies, with possible underlying mechanisms including increased platelet aggregation, AP drug-induced sedation leading to venostasis and clot formation [64]. APs are also thought to be associated with insulin resistance and weight gain, which in turn are risk factors for cardio- and cerebro-vascular disease [65].

Our review benefits from a number of strengths: we identified studies using a detailed and comprehensive search strategy; we performed meta-analyses using a carefully considered approach which took due account of important methodological differences between studies, including study design and type of effect estimate (whereas previous meta-analyses have been less careful in their meta-analytical approach); and we assessed individual study methodological quality, paying particular attention to the potential for confounding in many studies, which previous reviews have rarely highlighted.

Our review has limitations, mainly due to shortcomings of individual studies and the challenges of meaningfully synthesizing such heterogeneous studies. Whilst selection and information bias were minimized through the use of routinely collected health datasets in almost all studies, confounding by indication is a major limitation, since having a major mental disorders is itself associated with increased risk of stroke and MI [5]. The excess risk of cardiovascular disease in people with major mental illness is thought to be multifactorial and to include poor lifestyle behaviours (such as smoking, alcohol misuse, physical inactivity and obesity), increased risk of diabetes, shared genetic factors and direct physiological effects of the mental illness [66–68]. Whilst APs might themselves increase the risk of cardiovascular disease, particularly through weight gain, it is interesting that metabolic dysregulation for example, has been observed in AP naïve people with major mental illness [69]. In the studies identified in our review confounders such as lifestyle factors and socioeconomic status were rarely adequately adjusted for. The definition of stroke varied considerably across studies, with some also including non-stroke cerebrovascular disease ICD codes and others adopting a very narrow definition of stroke. Finally, studies were also heterogeneous in their definition of AP drug use and duration of follow-up varied widely, from just weeks to 13 years.

Further research is needed to address the gaps identified in this review. For ethical reasons, RCTs in this area are less feasible than observational studies, since, for some conditions, there are no suitable potential alternatives to AP drug treatment. However, where potential alternative treatments do exist, RCTs may be appropriate. In the dementia population for example, future studies could examine how the risk of stroke and MI amongst AP drug users compares to alternative treatment for behavioural and psychotic symptoms. For example, findings from a cohort study conducted by Finkel may suggest that AP drug use has a potentially lower risk of stroke compared to the use of benzodiazepines in dementia patients [70]. Where RCTs are not feasible, robust cohort and case-control studies should be performed. Such studies should be sufficiently large in order to be adequately powered to detect significant associations with high precision. Pooling of individual patient data across multiple settings would be one approach to enhance study power. Routinely collected national health data which contains information on mental health diagnosis and treatment, previous and subsequent cardiovascular events and confounding factors would be the ideal data source for future studies. This relies on the existence of robust mental health registries linked to other health data or linkage of primary and secondary care data as well as prescription data, which exists in relatively few settings. However, such studies would eliminate bias, facilitate control of confounders and establish temporality between AP drug use and cardiovascular outcome, particularly in case-control studies. Future studies should also distinguish between FGA and SGA users, given their differing pharmacological properties and potentially different risk of stroke and MI. Confounding by indication should be minimized and risk estimates should be determined based on indication for AP drug use. Studies should also assess the risk over multiple time periods in order to clarify whether and how the association may change with time. A minimum duration of AP use should also be clearly established (such as multiple prescriptions) especially if large registry databases are used to ascertain exposure.

In the absence of conclusive evidence that AP drug use increases risk of stroke or MI, we recommend that clinicians should carefully weigh the potential benefits and risks of AP drug use on an individual basis, and evaluate cardiovascular risk prior to AP drug initiation and during treatment. We urge clinical caution in the initiation of off-label AP drug use and encourage the exploration of alternative treatment routes.

## Conclusions

In conclusion, there is some evidence that AP drug use is associated with an increased risk of stroke, but no clear evidence that it is associated with an increased risk of MI. The risk of stroke was less apparent in studies specifically reporting on a population with dementia. However, these conclusions are drawn with the caveat that there was substantial methodological and statistical heterogeneity between studies and we highly recommend further methodologically robust studies which control for confounding factors, account for confounding by indication and examine associations by AP drug type and exposure period. Whilst further research is undertaken, psychiatrists and other physicians prescribing antipsychotic medications to people with or without a major mental disorder should be vigilant in monitoring and improving their cardiovascular risk profile.

## Additional file

Additional file 1: Supplemental Material. (DOCX 61 kb)

## Data Availability

Not applicable.

## Abbreviations

AP: Antipsychotic
CI: Confidence interval
FGA: First-generation antipsychotic
HR: Hazard ratio
IRR: Incident rate ratio
MI: Myocardial infarction
OR: Odds ratio
SGA: Second-generation antipsychotic
