# Supplementary material for: Antipsychotic drug use and risk of stroke and myocardial infarction: a systematic review and meta-analysis
Source: BMC Psychiatry. 2019 Jun 20;19:189. doi: 10.1186/s12888-019-2177-5 (PMC6585081; doi:10.1186/s12888-019-2177-5)
Supplement: Supplementary file 1 — Supplemental Material. (DOCX 61 kb) [file 12888_2019_2177_MOESM1_ESM.docx]

**eSupplement 1 Electronic search strategy, for Embase, Medline, PsychInfo and Cochrane Database**

## Embase Search Strategy

1. neuroleptic agent/ or exp atypical antipsychotic agent/ or exp chlorpromazine/ or exp chlorpromazine sulfoxide/ or exp clopenthixol/ or exp clopenthixol decanoate/ or exp droperidol/ or exp flupentixol/ or exp flupentixol decanoate/ or exp fluphenazine/ or exp fluphenazine decanoate/ or exp fluphenazine enanthate/ or exp haloperidol/ or exp haloperidol decanoate/ or exp loxapine/ or exp loxapine succinate/ or exp perphenazine/ or exp perphenazine decanoate/ or exp perphenazine enanthate/ or exp pimozide/ or exp prochlorperazine/ or exp prochlorperazine edisylate/ or exp prochlorperazine maleate/ or exp promazine/ or exp reduced haloperidol/ or exp thioridazine/ or exp trifluoperazine/ or exp trifluoperazine derivative/ or exp zuclopenthixol/ or exp zuclopenthixol acetate/ or exp zuclopenthixol decanoate/
2. dopamine receptor blocking agent/
3. psychotropic agent/
4. (haloperidol or chlorpromazine or fluphenazine or flupenthixol or loxapine or perphenazine or pimozide or clopenthixol or zuclopenthixol or levomepromazine or pericyazine or promazine or sulpiride or prochlorperazine or droperidol or thiothixene or thioridazine or trifluoperazine or aripiprazole or clozapine or paliperidone or risperidone or quetiapine or amisulpride or lurasidone or iloperidone or asenapine or olanzapine or ziprasidone).mp. [mp=title, abstract, heading word, drug trade name, original title, device manufacturer, drug manufacturer, device trade name, keyword]
5. (antipsychotic$ or psychotrop$ or neuroleptic$ or dopamine receptor block$ or dopamine receptor antagonist$).mp. [mp=title, abstract, heading word, drug trade name, original title, device manufacturer, drug manufacturer, device trade name, keyword]
6. 1 or 2 or 3 or 4 or 5
7. exp cerebrovascular accident/ or cerebrovascular disease/
8. exp brain hemorrhage/
9. exp brain infarction/
10. exp ischemic heart disease/
11. (stroke or intracranial hemorrhage or cerebrovascular accident$ or myocardial infarct$ or coronary artery disease or coronary heart disease or coronary disease or ischemic heart disease or ischaemic heart disease).mp. [mp=title, abstract, heading word, drug trade name, original title, device manufacturer, drug manufacturer, device trade name, keyword]
12. 7 or 8 or 9 or 10 or 11
13. 6 and 12
14. limit 13 to (human and english language)



   

 ! "  

  ## $#! %$&#

!   #!'!($)%) $$) !*

% +)%, +)&+ +)$

$!  +)$!  +) -

 ./

0 $$$!1 .$ 1 .$ $$ 

 1 !1$ $1$ $

$2!!1 1 !1 $$1 

$ 1 .$1 1$

$1 $  - !$

$ $   $ 1 1 !

'!($)%) $$) !*% +)%, 

+)&+ +)$$!  +)

$!  +) - ./

3 0

4 %2$% !  $

!&

5   ! !$

 * 

6 &  $!%2$ #!$

 *#   

!!!'!($)

%) $$) !*% +)%, +)&+

 +)$$!  +)

$!  +) - ./

7 456

 3 7

 $!! 

 $! $$ 



   

 ! "  

  ## $#! %$&#

!   #!'!($)%) $$) !*

% +)%, +)&+ +)$

$!  +)$!  +) -

 ./

0 $$$!1 .$ 1 .$ $$ 

 1 !1$ $1$ $

$2!!1 1 !1 $$1 

$ 1 .$1 1$

$1 $  - !$

$ $   $ 1 1 !

'!($)%) $$) !*% +)%, 

+)&+ +)$$!  +)

$!  +) - ./

3 0

4 %2$% !  $

!&

5   ! !$

 * 

6 &  $!%2$ #!$

 *#   

!!!'!($)

%) $$) !*% +)%, +)&+

 +)$$!  +)

$!  +) - ./

7 456

 3 7

 $!! 

 $! $$ 

## MEDLINE Search Strategy

1. psychotropic drugs/ or exp antipsychotic agents/
2. exp Dopamine Antagonists/
3. (antipsychotic$ or psychotrop$ or neuroleptic$ or dopamine receptor block$ or dopamine receptor antagonist$).mp. [mp=title, abstract, original title, name of substance word, subject heading word, keyword heading word, protocol supplementary concept word, rare disease supplementary concept word, unique identifier]
4. (haloperidol or chlorpromazine or fluphenazine or flupenthixol or loxapine or perphenazine or pimozide or clopenthixol or zuclopenthixol or levomepromazine or pericyazine or promazine or sulpiride or prochlorperazine or droperidol or thiothixene or thioridazine or trifluoperazine or aripiprazole or clozapine or paliperidone or risperidone or quetiapine or amisulpride or lurasidone or iloperidone or asenapine or olanzapine or ziprasidone).mp. [mp=title, abstract, original title, name of substance word, subject heading word, keyword heading word, protocol supplementary concept word, rare disease supplementary concept word, unique identifier]
5. 1 or 2 or 3 or 4
6. cerebrovascular disorders/ or exp brain ischemia/ or exp intracranial hemorrhages/ or exp stroke/
7. exp acute coronary syndrome/ or exp coronary disease/ or exp myocardial infarction/
8. (stroke or intracranial hemorrhage or cerebrovascular accident$ or myocardial infarct$ or coronary artery disease or coronary heart disease or coronary disease or ischemic heart disease or ischaemic heart disease).mp. [mp=title, abstract, original title, name of substance word, subject heading word, keyword heading word, protocol supplementary concept word, rare disease supplementary concept word, unique identifier]
9. 6 or 7 or 8
10. 5 and 9
11. limit 10 to (humans and english language)

## PsychINFO Search Strategy

1. exp neuroleptic drugs/
2. exp dopamine antagonists/
3. (antipsychotic$ or psychotrop$ or neuroleptic$ or dopamine receptor block$ or dopamine receptor antagonist$).mp. [mp=title, abstract, heading word, table of contents, key concepts, original title, tests & measures]
4. (haloperidol or chlorpromazine or fluphenazine or flupenthixol or loxapine or perphenazine or pimozide or clopenthixol or zuclopenthixol or levomepromazine or pericyazine or promazine or sulpiride or prochlorperazine or droperidol or thiothixene or thioridazine or trifluoperazine or aripiprazole or clozapine or paliperidone or risperidone or quetiapine or amisulpride or lurasidone or iloperidone or asenapine or olanzapine or ziprasidone).mp. [mp=title, abstract, heading word, table of contents, key concepts, original title, tests & measures]
5. 1 or 2 or 3 or 4
6. cerebrovascular disorders/ or exp cerebral hemorrhage/ or exp cerebral ischemia/ or exp cerebrovascular accidents/ or exp subarachnoid hemorrhage/
7. heart disorders/ or exp coronary thromboses/ or exp myocardial infarctions/
8. (stroke or intracranial hemorrhage or cerebrovascular accident$ or myocardial infarct$ or coronary artery disease or coronary heart disease or coronary disease or ischemic heart disease or ischaemic heart disease).mp. [mp=title, abstract, heading word, table of contents, key concepts, original title, tests & measures]
9. 6 or 7 or 8
10. 5 and 9
11. limit 10 to (human and english language)

## The Cochrane Database Search Strategy

#1 MeSH descriptor: [Dopamine Antagonists] explode all trees

#2 MeSH descriptor: [Antipsychotic Agents] explode all trees

#3 MeSH descriptor: [Psychotropic Drugs] this term only

#4 antipsychotic* or psychotrop* or neuroleptic* or dopamine receptor block* or dopamine receptor antagonist*

#5 haloperidol or chlorpromazine or fluphenazine or flupenthixol or loxapine or perphenazine or pimozide or clopenthixol or zuclopenthixol or levomepromazine or pericyazine or promazine or sulpiride or prochlorperazine or droperidol or thiothixene or thioridazine or trifluoperazine or aripiprazole or clozapine or paliperidone or risperidone or quetiapine or amisulpride or lurasidone or iloperidone or asenapine or olanzapine or ziprasidone

#6 #1 or #2 or #3 or #4 or #5

#7 MeSH descriptor: [Stroke] explode all trees

#8 MeSH descriptor: [Intracranial Hemorrhages] this term only

#9 MeSH descriptor: [Coronary Disease] explode all trees

#10 MeSH descriptor: [Acute Coronary Syndrome] explode all trees

#11 MeSH descriptor: [Brain Ischemia] explode all trees

#12 stroke or intracranial hemorrhage or cerebrovascular accident* or myocardial infarct* or coronary artery disease or coronary heart disease or coronary disease or ischemic heart disease or ischaemic heart disease

#13 #7 or #8 or #9 or #10 or #11 or #12

#14 #6 and #13

**eTable 1 Quality assessment of included studies reporting on antipsychotic drug use and risk of stroke**

|  | **Aim** | **Selection of Participants** | | | | **Assessment** | | | **Confounding** | **Chance** |
| --- | --- | --- | --- | --- | --- | --- | --- | --- | --- | --- |
| **Study** | **Clear and focused**  **study question** | **Comparable source**  **populations used to**  **identify participants**  **in 2 groups** | **Reported baseline**  **characteristics**  **between comparison groups** | **Reported**  **Participation rate**  **(loss to follow up)** | **Comparison in**  **baseline**  **characteristics**  **between participants and non-participants** | **Assessment of**  **Exposure valid** | **Outcome (cases)**  **clearly defined** | **Outcome**  **ascertainment valid** | **Key confounders**  **accounted for**  **in design and**  **analysis** | **Confidence intervals reported alongside**  **effect estimate** |
| Barnett, 2007^28^ | ☺ | ☺ | ☺ | NA | NA | 😐 | ☺ | 😐 | 😐 | ☺ |
| Chan, 2010^29^ | ☺ | ☺ | ☺ | NA | NA | 😐 | ☺ | 😐 | 😐 | ☺ |
| Chen, 2008^30^ | ☺ | ☺ | ☺ | NA | NA | 😐 | ☺ | 😐 | ☺ | ☺ |
| Correll, 2015^31^ | ☺ | ☺ | ☺ | NA | NA | 😐 | ☺ | 😐 | 😐 | ☺ |
| Douglas, 2008^32^ | ☺ | NA | 😐 | NA | NA | ☺ | ☺ | ☺ | ☺ | ☺ |
| Franchi, 2013^33^ | ☺ | ☺ | ☺ | NA | NA | 😐 | ☺ | 😐 | 😐 | ☺ |
| Hsieh, 2013^34^ | ☺ | ☺ | ☺ | NA | NA | ☺ | ☺ | ☺ | 😐 | ☺ |
| Kleijer, 2009^35^ | ☺ | ☺ | ☺ | NA | NA | 😐 | ☺ | 😐 | 😐 | ☺ |
| Lan, 2015^36^ | ☺ | ☺ | ☹ | NA | NA | 😐 | ☺ | 😐 | 😐 | ☺ |
| Laredo, 2011^37^ | ☺ | ☺ | ☺ | NA | NA | 😐 | ☺ | ☺ | 😐 | ☺ |
| Liebetrau, 2008^38^ | ☺ | ☺ | ☹ | ☺ | ☺ | ☹ | ☺ | ☺ | 😐 | ☺ |
| Liperoti, 2005^39^ | ☺ | ☺ | ☺ | NA | NA | 😐 | ☺ | ☺ | ☺ | ☺ |
| Liu, 2013^40^ | ☺ | ☺ | ☹ | NA | NA | 😐 | ☺ | 😐 | ☹ | ☺ |
| Percudani, 2005^41^ | ☺ | ☺ | ☹ | ☹ | ☹ | 😐 | ☺ | 😐 | ☹ | ☺ |
| Pratt, 2010^42^ | ☺ | NA | 😐 | NA | NA | 😐 | ☺ | 😐 | ☺ | ☺ |
| Sacchetti, 2008^43^ | ☺ | ☺ | ☺ | NA | NA | 😐 | ☺ | ☺ | ☺ | ☺ |
| Shin, 2013^44^ | ☺ | NA | ☹ | NA | NA | 😐 | ☺ | ☺ | ☺ | ☺ |
| Wang, 2012^45^ | ☺ | NA | ☺ | NA | NA | 😐 | ☺ | ☺ | ☺ | ☺ |
| Wu, 2012^46^ | ☺ | NA | ☺ | NA | NA | 😐 | ☺ | 😐 | ☺ | ☺ |

☺ = Yes; ☹ = Failed to report or failed to validate self reported information with a second source; 😐 = Reported, however may be incomplete or, for exposure and outcome ascertainment, validation/accuracy of data used not provided; NA = not applicable

**eTable 2 Quality assessment of included studies reporting on antipsychotic drug use and risk of myocardial infarction**

|  | **Aim** | **Selection of Participants** | | | | **Assessment** | | | **Confounding** | **Chance** |
| --- | --- | --- | --- | --- | --- | --- | --- | --- | --- | --- |
| **Study** | **Clear and focused**  **study question** | **Comparable source**  **populations used to**  **identify participants**  **in 2 groups** | **Reported baseline**  **characteristics**  **between comparison groups** | **Reported**  **Participation rate**  **(loss to follow up)** | **Comparison in**  **baseline**  **characteristics**  **between participants and non-participants** | **Assessment of**  **Exposure is valid** | **Outcome (cases) clearly defined** | **Outcome**  **ascertainment**  **valid** | **Key confounders**  **accounted for**  **in design and**  **analysis** | **Confidence intervals reported alongside**  **effect estimate** |
| Brauer, 2015^47^ | ☺ | ☺ | ☺ | NA | NA | 😐 | ☺ | ☺ | 😐 | ☺ |
| Correll, 2015^31^ | ☺ | ☺ | ☺ | NA | NA | 😐 | ☺ | 😐 | 😐 | ☺ |
| Enger, 2004^48^ | ☺ | ☺ | ☺ | NA | NA | 😐 | ☺ | 😐 | ☹ | ☺ |
| Hwang, 2014^49^ | ☺ | ☺ | ☺ | NA | NA | ☺ | ☺ | ☺ | ☺ | ☺ |
| Lin, 2014^50^ | ☺ | NA | ☺ | NA | NA | 😐 | ☺ | ☺ | ☺ | ☺ |
| Nakawaga, 2006^51^ | ☺ | ☺ | ☺ | NA | NA | 😐 | ☺ | ☺ | ☹ | ☺ |
| Pariente, 2012^52^ | ☺ | ☺ | ☺ | NA | NA | 😐 | ☺ | 😐 | 😐 | ☺ |
| Penttinen, 1996^53^ | ☺ | ☺ | ☹ | ☺ | ☹ | 😐 | ☺ | ☺ | ☹ | ☺ |
| Pratt, 1996^54^ | ☺ | ☺ | ☹ | ☺ | ☹ | ☹ | ☺ | ☹ | ☹ | ☺ |
| Wang, 2011^55^ | ☺ | NA | ☹ | NA | NA | 😐 | ☺ | 😐 | ☺ | ☺ |

☺ = Yes; ☹ = Failed to report or failed to validate self reported information with a second source; 😐 = Reported, however may be incomplete or, for exposure and outcome ascertainment, validation/accuracy of data used not provided; NA = not applicable

**eTable 3 Factors adjusted for, or matched on, in each identified study reporting on antipsychotic drug use and stroke**

| **Study author** | **Study design** | **Mental and physical disease history** | | | | | | | | | | **Medications** | | **Lifestyle** | | | | **Sociodemographics** | | | | | **Additional factors adjusted for/matched on** |
| --- | --- | --- | --- | --- | --- | --- | --- | --- | --- | --- | --- | --- | --- | --- | --- | --- | --- | --- | --- | --- | --- | --- | --- |
|  |  | **Diagnosis of Schizophrenia** | **Diagnosis of mood disorder** | **Other mental health disorder** | **Dementia** | **CVD** | **Previous stroke** | **Diabetes** | **Hypertension\BP** | **Number of medical comorbidities/com-orbidity index** | **dyslipidaemia** | **Cardiovascular medications** | **Other psychiatric medications** | **Smoking** | **Alcohol** | **BMI/obesity** | **Physical activity** | **Age** | **Sex** | **Race** | **Socioeconomic status** | **Marital status** |  |
| Barnett, 2007^28^ | Cohort | 😐 | 😐 | 😐 | NA | 😐 | ☺ | ☺ | ☺ | ☺ | ☺ | ☺ | ☹ | ☹ | ☹ | ☹ | ☹ | ☺ | ☺ | ☺ | ☹ | ☺ | VA status and dementia type |
| Chan, 2010^29^ | Cohort | NA | 😐 | ☹ | NA | ☺ | ☺ | ☺ | ☺ | ☹ | ☺ | 😐 | 😐 | ☺ | ☹ | ☹ | ☹ | ☺ | ☺ | ☹ | ☺ | ☺ | Residence, dementia type, malignant neoplasm, and number of drugs prescribed |
| Chen, 2008^30^ | Nested case-control | ☺ | ☺ | ☺ | ☹ | ☺ | NA | ☺ | ☺ | ☹ | ☺ | ☺ | ☺ | ☹ | ☺ | ☺ | ☹ | ☺ | ☺ | ☹ | ☹ | ☹ | Year of depression diagnosis |
| Correll, 2015^31^ | Cohort | ☺ | ☺ | ☹ | ☹ | NA | NA | NA | NA | ☺ | NA | NA | ☹ | ☹ | ☹ | NA | ☹ | ☺ | ☺ | ☹ | ☹ | ☹ |  |
| Douglas, 2008^32^ | Self-controlled case series | ☺ | ☺ | ☺ | ☺ | ☺ | NA | ☺ | ☺ | ☺ | ☺ | ☺ | ☺ | 😐 | 😐 | 😐 | 😐 | ☺ | ☺ | ☺ | 😐 | 😐 | Age at time of stroke; acted as own control for confounders |
| Franchi, 2013^33^ | Case-control | 😐 | 😐 | 😐 | 😐 | 😐 | 😐 | 😐 | ☺ | ☹ | ☺ | ☺ | ☹ | ☹ | ☹ | ☹ | ☹ | ☺ | ☺ | ☹ | ☹ | ☹ | Also matched on local health unit and adjusted for dementia medications |
| Hsieh, 2013^34^ | Nested case-control | NA | ☹ | ☹ | ☺ | ☺ |  | ☺ | ☺ | ☹ | ☺ | ☺ | ☺ | ☹ | ☹ | ☹ | ☹ | ☺ | ☺ | ☹ | ☹ | ☹ | COPD, parkinsonism and neoplasm |
| Kleijer, 2009^35^ | Nested case-control | ☹ | ☹ | ☹ | ☹ | 😐 | 😐 | 😐 | 😐 | ☹ | ☺ | ☺ | ☹ | ☹ | ☹ | ☹ | ☹ | ☺ | ☺ | ☹ | ☹ | ☹ |  |

**eTable 3 Factors adjusted for, or matched on, in each identified study reporting on antipsychotic drug use and stroke (continued)**

| **Study author** | **Study design** | **Mental and physical disease history** | | | | | | | | | | **Medications** | | **Lifestyle** | | | | **Sociodemographics** | | | | | **Additional factors adjusted for/matched on** |
| --- | --- | --- | --- | --- | --- | --- | --- | --- | --- | --- | --- | --- | --- | --- | --- | --- | --- | --- | --- | --- | --- | --- | --- |
|  |  | **Diagnosis of Schizophrenia** | **Diagnosis of mood disorder** | **Other mental health disorder** | **Dementia** | **CVD** | **Previous stroke** | **Diabetes** | **Hypertension\BP** | **Number of medical comorbidities/com-orbidity index** | **dyslipidaemia** | **Cardiovascular medications** | **Other psychiatric medications** | **Smoking** | **Alcohol** | **BMI/obesity** | **Physical activity** | **Age** | **Sex** | **Race** | **Socioeconomic status** | **Marital status** |  |
| Lan, 2015^36^ | Cohort | ☹ | NA | ☹ | ☹ | ☺ |  | ☺ | ☺ | ☹ | ☺ | ☹ | ☺ | ☹ | ☹ | ☹ | ☹ | ☺ | ☺ | ☹ | ☹ | ☹ | Kidney disease and use of other AP class |
| Laredo, 2011^37^ | Nested case-control | ☹ | ☹ | ☹ | NA | ☺ | NA | ☺ | ☺ | ☹ | ☺ | ☺ | ☹ | ☹ | ☹ | ☺ | ☹ | ☺ | ☺ | ☹ | ☹ | ☹ |  |
| Liebetrau, 2008^38^ | Cohort | ☹ | ☹ | ☺ | ☺ | ☹ | NA | ☹ | ? | ☹ | ☹ | ☹ | NA | ☹ | ☹ | ☹ | ☹ | ☺ | ☺ | ☹ | ☹ | ☹ |  |
| Liperoti, 2005^39^ | Case-control | NA | ☺ | ☺ | ☺ | ☺ | ☺ | ☺ | ☺ | ☹ | ☺ | ☺ | 😐 | ☹ | ☹ | ☺ | ☹ | ☺ | ☺ | ☺ | ☹ | ☹ | Matched on nursing care facility and time period; adjusted for functional and cognitive status, |
| Liu, 2013^40^ | Cohort | ☹ | ☹ | ☹ | ☹ | ☹ | NA | ☺ | ☺ | ☹ | ☹ | ☹ | ☹ | ☹ | ☹ | ☹ | ☹ | ☺ | ☺ | ☹ | ☺ | ☹ | Geographical regions |
| Percudani2005^41^ | Cohort | ☹ | ☹ | ☹ | ☹ | ☹ | ☹ | ☹ | ☹ | ☹ | ☹ | 😐 | 😐 | ☹ | ☹ | ☹ | ☹ | ☺ | ☺ | ☹ | ☹ | ☹ | Number of antipsychotic prescriptions |
| Pratt, 2010^42^ | Self-controlled case series | ☺ | ☺ | ☺ | ☺ | ☺ | ☺ | ☺ | ☺ | ☺ | ☺ | ☺ | ☺ | 😐 | 😐 | 😐 | 😐 | ☺ | ☺ | ☺ | 😐 | 😐 | Adjusted for calendar year |
| Sacchetti, 2008^43^ | Cohort | 😐 | 😐 | 😐 | 😐 | ☺ | NA | ☺ | ☺ | ☺ | ☺ | ☺ | 😐 | ☹ | ☹ | ☺ | ☹ | ☺ | ☺ | ☹ | ☹ | ☹ | Adjusted for psychiatric indication for antipsychotic drug use, benzodiazapines, COPD, recent history of pneumonia, malignant neoplasm |
| Shin, 2013^44^ | Case-crossover | ☺ | ☺ | ☺ | ☺ | ☺ | ☺ | ☺ | ☺ | ☺ | 😐 | ☺ | ☺ | 😐 | 😐 | 😐 | 😐 | ☺ | ☺ | ☺ | 😐 | 😐 |  |

**eTable 3 Factors adjusted for, or matched on, in each identified study reporting on antipsychotic drug use and stroke (continued)**

| **Study author** | **Study design** | **Mental and physical disease history** | | | | | | | | | | **Medications** | | **Lifestyle** | | | | **Sociodemographics** | | | | | **Additional factors adjusted for/matched on** |
| --- | --- | --- | --- | --- | --- | --- | --- | --- | --- | --- | --- | --- | --- | --- | --- | --- | --- | --- | --- | --- | --- | --- | --- |
|  |  | **Diagnosis of Schizophrenia** | **Diagnosis of mood disorder** | **Other mental health disorder** | **Dementia** | **CVD** | **Previous stroke** | **Diabetes** | **Hypertension\BP** | **Number of medical comorbidities/como-rbidity index** | **dyslipidaemia** | **Cardiovascular medications** | **Other psychiatric medications** | **Smoking** | **Alcohol** | **BMI/obesity** | **Physical activity** | **Age** | **Sex** | **Race** | **Socioeconomic status** | **Marital status** |  |
| Wang, 2012^45^ | Case-case-time-control | ☺ | ☺ | ☺ | ☺ | ☺ | ☺ | ☺ | ☺ | ☺ | ☺ | 😐 | 😐 | 😐 | 😐 | 😐 | 😐 | ☺ | ☺ | ☺ | 😐 | 😐 | Adjusted for time variant within person confounders and exposure time trends |
| Wu, 2012^46^ | Case crossover | ☺ | ☺ | ☺ | ☺ | ☺ | ☺ | ☺ | ☺ | ☺ | ☺ | ☺ | 😐 | 😐 | 😐 | 😐 | 😐 | ☺ | ☺ | ☺ | 😐 | 😐 | Adjusted for number of outpatient visits and CVD medication use |

☺ = potential confounder adjusted for; ☹ = potential confounder not adjusted for; 😐 = potential confounder probably or partially adjusted for; ? = unclear whether potential confounder adjusted for

NA = not applicable (generally because participants were excluded on the basis of the confounder in question)

**eTable 4 Factors adjusted for, or matched on, in each identified study reporting on myocardial infarction**

| **Study** | **Study design** | **Mental and physical disease history** | | | | | | | | | | | **Medications** | | **Lifestyle** | | | | **Sociodemographics** | | | | | **Additional factors adjusted for/matched on** |
| --- | --- | --- | --- | --- | --- | --- | --- | --- | --- | --- | --- | --- | --- | --- | --- | --- | --- | --- | --- | --- | --- | --- | --- | --- |
|  |  | **Diagnosis of schizophrenia** | **Diagnosis of mood disorders** | **Other mental health disorder** | **Dementia** | **History of angina** | **previous MI** | **Previous stroke** | **Diabetes** | **Hypertension\BP** | **Number of medical comorbidities** | **dyslipidaemia** | **Cardiovascular medications** | **Other psychiatric medications** | **Smoking** | **Alcohol** | **Weight\obesity** | **Physical activity** | **Age** | **Sex** | **Race** | **Socioeconomic status** | **Marital status** |  |
| Brauer, 2015^47^ | Case-control | ☹ | ☹ | ☹ | ☹ | ☺ | NA | ☺ | ☺ | ☺ | ☹ | ☺ | ☺ | ☹ | ☺ | ☺ | ☺ | ☹ | ☺ | ☺ | ☹ | ☹ | ☹ | Also matched by general practitioner |
| Correll, 2015^31^ | Cohort | ☺ | ☺ | ☺ | ☹ | NA | NA | NA | NA | NA | ☺ | NA | NA | ☹ | ? | ? | NA | ☹ | ☺ | ☺ | ☹ | ☹ | ☹ |  |
| Enger, 2004^48^ | Matched cohort | NA | ☹ | ☹ | NA | ? | ? | ? | ☺ | ? | ? | ☹ | ☺ | ☹ | ☹ | ☹ | ☹ | ☹ | ☺ | ☺ | ☹ | ☹ | ☹ | Index year, geographic location |
| Hwang, 2014^49^ | Matched cohort | ☺ | ☺ | ☺ | ☺ | ☺ | ? | ☺ | ☺ | ☺ | ☺ | ☺ | ☺ | ☺ | ☹ | ☺ | ☺ | ☹ | ☺ | ☺ | ☹ | ☺ | ☹ | Location, residential status (community-dwelling vs long-term care), Parkinson disease, chronic kidney disease |
| Lin, 2014^50^ | Case crossover | ☺ | ☺ | ☺ | ☺ | ☺ | ☺ | ☺ | ☺ | ☺ | ☺ | ☺ | ☺ | ☺ | 😐 | 😐 | 😐 | 😐 | ☺ | ☺ | ☺ | 😐 | 😐 | Adjusted for number of outpatient visits |
| Nakawaga, 2006^51^ | Case-control | ☹ | ☹ | ☹ | ☹ | ☹ | NA | ☺ | ☺ | ☺ | ☹ | ☺ | ☺ | ☹ | ☹ | ☹ | ☹ | ☹ | ☺ | ☺ | ☹ | ☹ | ☹ | Also matched on county of residence; adjusted for chronic bronchitis |
| Pariente, 2012^52^ | Matched cohort | ☹ | ☹ | ☹ | NA | ☺ | ☺ | ☺ | ☺ | ☺ | ☹ | ☺ | ☺ | ☺ | ☹ | ☹ | ☹ | ☹ | ☺ | ☺ | ☹ | ☹ | ☹ |  |
| Penttinen, 1996^53^ | Nested case-control | ☹ | ☹ | ☹ | ☹ | ☹ | NA | ☹ | ☹ | ☹ | ☹ | ☹ | ☹ | ☹ | ☺ | ☹ | ☹ | ☹ | ☺ | NA | ☹ | ☺ | ☹ | Also matched on county |
| Pratt, 1996^54^ | Cohort | ☹ | ☹ | ☺ | ☹ | ☹ | NA | ☹ | ☹ | ☺ | ☹ | ☹ | ☹ | ☹ | ☹ | ☹ | ☹ | ☹ | ☺ | ☺ | ☹ | ☹ | ☺ | Adjusted for dysphoria |
| Wang, 2011^55^ | Case- case-time control | ☺ | ☺ | ☺ | ☺ | ☺ | ☺ | ☺ | ☺ | ☺ | ☺ | ☺ | ☺ | ☺ | 😐 | 😐 | 😐 | 😐 | ☺ | ☺ | ☺ | 😐 | 😐 |  |

☺ = potential confounder adjusted for; ☹ = potential confounder not adjusted for; 😐 = potential confounder probably or partially adjusted for; ? = unclear whether potential confounder adjusted for

NA = not applicable (generally because participants were excluded on the basis of the confounder in question)
